# Natriuretic peptides testing and survival prediction models for chronic heart failure: a systematic review of added prognostic value

**DOI:** 10.1186/s41512-025-00210-x

**Published:** 2025-12-09

**Authors:** Charlotte A. Smith, Kathryn S. Taylor, Nicholas R. Jones, Dominik Roth, Amy Magona, Nia Roberts, Clare J. Taylor, F. D. Richard Hobbs, Maria D. L. A. Vazquez-Montes

**Affiliations:** 1 Royal Devon University Healthcare NHS Foundation Trust, Royal Devon and Exeter Hospital, Exeter, UK; 2 Nuffield Department of Primary Care Health Sciences, University of Oxford, Oxford, UK; 3 Department of Emergency Medicine, Medical University of Vienna, Vienna, Austria; 4 Oxford Clinical Trials Research Unit, Nuffield Department of Orthopaedics, Rheumatology and Musculoskeletal Sciences, University of Oxford, Oxford, UK; 5 Bodleian Health Care Libraries, University of Oxford, Oxford, UK; 6 Institute of Applied Health Research, University of Birmingham, Birmingham, UK

**Keywords:** B-type natriuretic peptide, N-terminal proBNP, Mortality, Prediction, Added value

## Abstract

**Background:**

High natriuretic peptide levels are associated with a poor outcome in adults with chronic heart failure (CHF). However, the incremented prediction accuracy of multivariable prognostic models after adding B-type natriuretic peptide (BNP) and/or N-terminal proBNP (NT-proBNP) remains unclear.

**Methods:**

We carried out a systematic review narrative analysis of added-value studies of BNP and NT-proBNP in CHF prognostication. Primary clinical studies investigating prognostic model development or validation in adult participants with CHF were included. Any studies of individual factors’ association with patient outcomes, treatment efficacy, or those using patients with transplant/ventricular assist devices, ≥ 10% of patients with advanced HF, or significant comorbidities, HF secondary to congenital/reversible conditions, or ≥ 33% of patients with valvular HF were excluded. The databases MEDLINE, Embase, Science Citation Index, and Cochrane Prognosis Methods Group Database were searched from January 1990 to February 2024. Predictive performance was measured in terms of discrimination and calibration, the added value in terms of the c-statistic difference before and after adding BNP and/or NT-proBNP to a base model, and the risk reclassification, namely, net reclassification index (NRI) and integrated discrimination improvement (IDI). Risk of bias assessment used the Prediction model Risk Of Bias ASsessment Tool (PROBAST).

**Results:**

Fourteen added-value studies comprising a total of 50,949 individuals were included. Both BNP and NT-proBNP consistently improved mortality prediction performance, but studies only presented separately before and after c-statistics, without formally testing for statistically significant differences. Meta-analysis was impossible due to missing data on the change in predictive performance and data heterogeneity. All studies reported discrimination. Few reported calibration, NRI, and IDI. All studies except one were deemed to be at high risk of bias, whereas 50% showed high applicability to the review question, with only 14% scoring high for applicability concern, and the rest were unclear.

**Conclusions:**

Improving consistency in researching and reporting the added value of natriuretic peptide testing to predict mortality in chronic heart failure patients could facilitate summarizing and interpreting the results more meaningfully.

**Registration:**

This review is a refinement of the methods and a search update of the review of added-value biomarkers in HF prognosis (PROSPERO registration number: CRD42019086993).

## Introduction

Heart failure (HF) is a common cause of mortality and morbidity [1]. As our population ages, the prevalence of HF is increasing, with around 64.3 million [2] individuals worldwide living with the condition. For those diagnosed with chronic heart failure (CHF), around 20% die within 1 year [3, 4], while 5-year survival rates for advanced HF are worse than those for some common cancers [5, 6].

Optimising treatment of HF at an early stage can improve patients’ quality of life and overall survival [7]. Prognostic models help patients and clinicians to make better-informed decisions regarding these treatments based on the risk of specified outcomes occurring within a given timeframe. Prognostic models can also inform discussions around lifestyle choices, advanced care planning, and help to set realistic expectations. The accuracy of a prognostic model is assessed in terms of its calibration (the model’s capacity to generate predicted probabilities similar to observed probabilities, usually evaluated graphically and/or using the ratio of observed to expected number of events) and discrimination (the ability of the model to correctly classify patients with and without the outcome of interest, measured by the c-statistic or, equivalently, the area under the receiver operating characteristic curve (AUC)). The accuracy across different populations (external validation studies) can be summarised with a systematic review and meta-analysis.

Despite the potential benefits of prognostic models, none are universally recommended by clinical guidelines for heart failure. For instance, none are recommended by NICE [8, 9], and even though the European Society of Cardiology’s (ESC) HF guidelines [10] suggest that risk models can help identify patients who are most suitable for advanced HF therapies, ESC is cautious about their recommendation of a few established models, including the Seattle Heart Failure Model (SHFM) [11] and Meta-analysis Global Group in Chronic Heart Failure (MAGGIC) model [12]. These two models and others are recommended by the AHA/ACC/HFSA guideline [13] for the management of HF and the ISHLT guideline [14] for cardiac transplantation candidates. However, prognostic scores are still not widely used in routine practice, with one study reporting only 1% of patients who received a prognostic estimate. In part, this may be due to concerns among clinicians about the accuracy of the scores, with previous studies demonstrating a tendency for scores to overestimate individuals’ risk [15].

There has been increasing interest in the added value to existing models of biomarkers with high prognostic power, such as high-sensitive cardiac troponin T (hs-cTnT) [16] and natriuretic peptides [17]. None of the established prognostic models includes these biomarkers, but they have started featuring in more recently developed models (such as natriuretic peptide in the LIFE-HF model [18]), which often lack an extensive external validation assessment. Natriuretic peptides (B-type natriuretic peptide (BNP) and N-terminal proBNP (NT-proBNP)) are central to the current HF diagnostic pathway, and they are known to have prognostic importance for HF outcomes including hospitalisation and death [19, 20]. BNP is a hormone secreted by cardiomyocytes in the heart ventricles and synthesised as a reaction to stress or distension. During secretion, a prohormone (proBNP) is split into the physiologically active BNP (32 amino acids) and the biologically inactive N-terminal fragment (NT-proBNP) [21]. The extent to which BNP and NT-proBNP might improve the accuracy of prognostic scores remains uncertain. A previous review of studies published up to 2012 summarised the incremental value of NT-proBNP in predicting mortality and morbidity of individuals with CHF [22]. This preceded the introduction of the risk of bias tool for prediction models [23], reporting guidelines for prediction models [24], and the publication of methods to synthesise performance data [25]. Our study aimed to implement the latest methodological developments in prognostic model research and provide an up-to-date summary of the added value of incorporating BNP and/or NT-proBNP into multivariable CHF prognostic models predicting mortality.

## Methods

This systematic review was reported in line with the recommendations from the Preferred Reporting Items for Systematic reviews and Meta-Analyses (PRISMA) statement [26] (Appendix 1). This review was a refinement of the methods and a search update of the review of added-value biomarkers in HF prognosis (PROSPERO registration number: CRD42019086993), which was itself part of a broad umbrella review [27]. Protocol amendments are reported in Appendix 2.

### Search strategy

The original umbrella review identified potential studies by searching MEDLINE (OvidSP) [1946–], Embase (OvidSP) [1974–], Science Citation Index (Web of Science Core Collection) [1900–], and the database of prognostic studies maintained by the Cochrane Prognosis Methods Group with no language restriction to 31 December 2019. The search started in 1990 when biomarkers, in particular natriuretic peptides, gained prominence as HF risk factors. Updates extended the search to 23 February 2024. Published search filters [28, 29] were combined for a sensitive search strategy with search terms to identify the added value of BNP and/or NT-proBNP to prognostic models for CHF (Appendix 3). The PICOTS (Population, Intervention, Comparator, Outcome, Timing, and Setting) in Table 1 set out the clinical question of this review.

**Table 1:** PICOTS for this systematic review

PICOTS element	Description
Population	Human adults aged 18 or over with a CHF diagnosis
Intervention (model)	A base model plus added BNP and/or NT-proBNP, where the base model is a multivariable model (two or more variables) for predicting the CHF clinical outcome mentioned below that does not contain BNP and/or NT-proBNP as prognostic factors. The purpose of the model must be to yield absolute risk probabilities for individual patients
Comparator	The base model
Outcome	All-cause mortality or a composite outcome that includes all-cause mortality
Timing	Prediction horizon of 1 year or greater. The BNP and/or NT-proBNP should be measured at baseline
Setting	No constraint, as this will vary between studies. Any setting delivering care to patients with CHF

The details of the systematic review’s question

*Abbreviations*: *BNP* B-type natriuretic peptide, *CHF* Chronic heart failure, *NT-proBNP* N-terminal pro-B-type natriuretic peptide, *PICOTS* Population, Intervention, Comparator, Outcome, Timing, and Setting

### Eligibility criteria and study screening

The inclusion and exclusion criteria are outlined in Table 2.

**Table 2:** Inclusion and exclusion criteria

Criteria	Type of studies	Target population
Inclusion	Only primary clinical studies of CHF with clinical models that present the following: Prognostic model development, validation, or updating with/without external validation considering added-value performance of at least one of BNP or NT-proBNP The sources of data could be medical records, existing RCT data, or large clinical databases	Adult patients 18+ years old, diagnosed with CHF irrespective of left ventricular ejection fraction. Those patients may or may not have already received optimum medical therapy, including medications and implantable devices (e.g. implantable cardioverter defibrillator and cardiac resynchronisation therapy devices)
Exclusion	Studies using exclusive assay analyses Studies published only as abstracts or clinical trials reporting no prognostic modelling on HF patients Studies developing models with the sole intention of evaluating the independent or adjusted association of a factor (even if this is a biomarker) with the outcome and not to predict individual probabilities Studies that explore the prognostic effect of treatment (e.g. medication regimes, device implantation) Systematic reviews, unless the authors use a review to form a data repository for developing a prognostic model. Their citation list will be explored for further inclusion of primary studies potentially missed by the sensitive search Literature reviews Case studies Diagnostic studies Studies focusing on economic evaluations of HF care	Patients who are recipients of, or already registered candidates for, transplantation or left/biventricular assist devices as their HF status will be significantly altered by this intervention At least 10% of the patients included have advanced/end-stage HF^*^ and/or are receiving end-of-life or palliative care or where a study only included patients with HF on the basis of having another comorbidity Patients with HF due to congenital conditions and secondary to reversible causes, e.g. pregnancy and peripartum, infection, major surgery, pre-revascularisation, intensive care conditions, and valvular disease (which has a maximum cut-off of 33.3% of patients to reflect the prevalence in the population) Patients with concomitant disease which predominantly affects prognosis, e.g. cancer and neurological disorders Patients with AHF

The 10% and 33.3% cut-off values were based on the clinical team’s expertise

*Abbreviations*: *AHF* Acute heart failure, *CHF* Chronic heart failure, *HF* Heart failure, *RCT* Randomised control trial

^*^Advanced HF is defined as New York Heart Association class IV HF

Records from the search were managed using EndNote 20 and screened by two independent reviewers, with disagreements resolved by discussion with a third reviewer. Initial screening involved reading only the title and abstract. The full texts of studies marked for inclusion or potential inclusion by at least one reviewer were subsequently read to assess eligibility.

### Data extraction

Data were extracted and verified by a second reviewer using a piloted extraction form based on the CHecklist for critical Appraisal and data extraction for systematic Reviews of prediction Modelling Studies (CHARMS) [30] and the Prediction model Risk Of Bias ASsessment Tool (PROBAST) [23]. Appendix 4 lists the data extracted.

### Risk of bias assessment

Risk of bias for individual publications was assessed independently by two reviewers using questions from PROBAST integrated into the extraction forms. Following PROBAST guidelines, the risk of bias for each study was rated as “low”, “high” or “unclear”, overall, and within four domains: participants, predictions, outcomes, and analysis. The same scoring system was used to judge the studies’ applicability to this review within the former domains, except for “analysis”. A risk of bias graph and table were produced using RobVis [31]. The certainty of evidence was not assessed, as there is currently no such tool for predictive models.

### Data analysis

Characteristics of the included studies and their populations were tabulated, reporting the mean and standard deviation of continuous variables and the number and percentage of categorical variables. Frequencies of missing values were reported for each relevant item.

Discrimination improvement was evaluated through the change in the c-statistic. We referred to the c-statistic throughout, although sometimes AUC was reported. When not reported, the change in c-statistic was calculated by subtracting the corresponding value before the addition of biomarker(s) from that recorded after biomarker addition. For these differences, 95% confidence intervals (95% CI) were not calculated. Risk reclassification was considered in terms of the net risk reclassification index (NRI) and the integrated discrimination improvement (IDI). Calibration was assessed using calibration plots or the Hosmer-Lemeshow test.

We planned to carry out random-effects meta-analysis with a formal analysis of between-study heterogeneity. As there were insufficient studies, we instead reported the results narratively, using summary tables and forest plots to present data. Homogeneity was assessed in terms of the study conduct, purpose and quality, base model, biomarkers, outcome definition, and the prediction horizon.

## Results

**Fig. 1:**
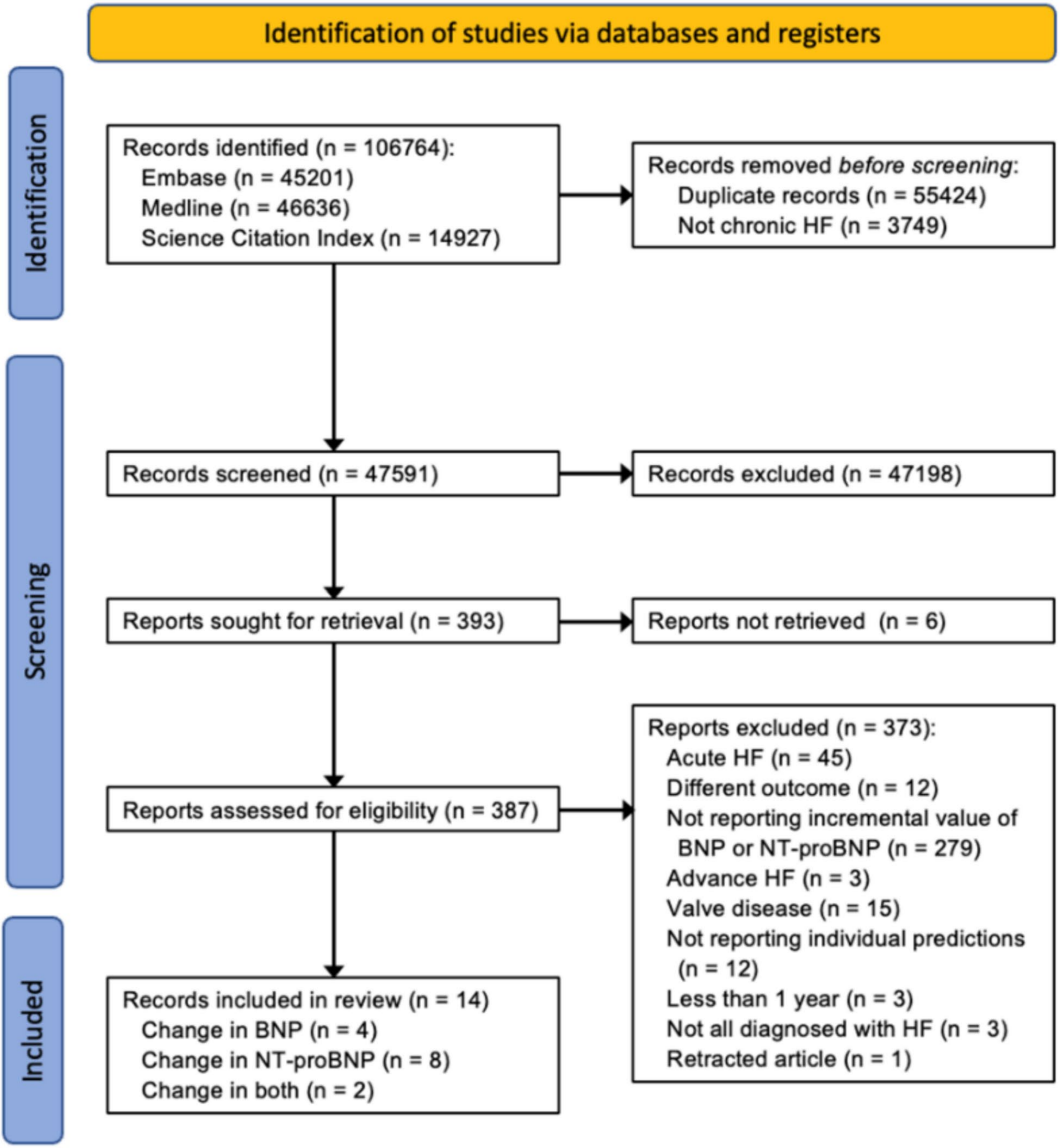
Adapted PRISMA flow diagram. Flow diagram showing the number of records included and excluded at each stage of the screening process after the initial search, with reasons for exclusion. Abbreviations: BNP, B-type natriuretic peptide; HF, heart failure; NT-proBNP, N-terminal pro-B-type natriuretic peptide

The search for the wider umbrella project identified 106,764 records [27] but incorporated both acute and chronic HF and omitted any restriction on the type of biomarker and prediction horizon. From these, we identified and screened 387 full texts, of which 373 were excluded, largely due to not having evaluated the incremental value of BNP or NT-proBNP, leaving 14 to be included in this review (Fig. 1).

### Study characteristics and model performance

Of the 14 included studies, 7 (50%) were conducted in the USA, with the rest spread across Europe. Data were reported for a total of 50,949 individuals across the study populations (range 54–7876). The participants were middle-to-old age on average (range of means 56–76 years), and of those reporting ethnicity, the proportion with white ethnicity ranged between 66 and 93%. All included studies, except Dunlay [32], had greater than 50% representation of males in their sample.

A range of prognostic outcomes was reported across the studies, for example all-cause mortality, left ventricular assist device implantation (LVAD), and heart transplantation. Where possible, data for all-cause mortality alone were extracted in preference to composite outcomes. Two studies [33, 34] only reported the composite outcomes all-cause mortality/heart transplant/LVAD and all-cause mortality/urgent heart transplant/LVAD, respectively. The prediction horizons varied, with 1 year as the most common, and some studies considered more than one prediction horizon [33, 35]. Only one study [33] reported a horizon of over 5 years (Table 3).

**Table 3:** Population and study characteristics

	Age, mean (SD)	Female, *n* (%)	Ethnicity, *n* (%); White	Asian	Black	Other	CHF type	Drugs: ACEi	Beta blocker	MRA	Diuretics	Population, *n*	Events, *n*	Outcome	Follow-up years, median (IQR)	Horizon, years	Country
AbouEzzedine (2016) [36]	69.0 (14.0)	123 (28)	410 (93)	0 (0)	9 (2)	22 (5)	HFrEF	√	√	-	√	441	75	M	1.0*	1.0	USA
Arzilli (2018) [37]	68.0 (12.0)	592 (25)	-	-	-	-	HFrEF	√	√	√	-	2368	198, 75	M	12.0*	1.0	Italy
Dunlay (2009) [32]	76.0 (12.8)	308 (52)	-	-	-	-	-	√	√	-	UC	593	122	M	-	1.0	USA
Dupuy (2019) [38]	75.7 (11.9)	51 (31)	-	-	-	-	-	√	√	-	√	164	63	M	3.5	3.5	France
Ky (2011) [39]	56.3 (14.0)	377 (33)	856 (75)	0 (0)	240 (21)	46 (4)	HFrEF	√	√	√	√	1141	267	M	2.8	5.0	USA
Ky (2012) [40]	56.0 (15.0)	514 (34)	1120 (74)	0 (0)	333 (22)	61 (4)	HFrEF	√	√	√	√	1513	137	M	2.5 (2.6)	1.0	USA
May (2007) [33]	67.0 (13.0)	1590 (39)	-	-	-	-	-	√	√	-	√	4077	2142	C	4.4* (4.2**)	1.0, 5.0	USA
McGranaghan (2020) [41]	72.0 (4.9)	73 (26)	-	-	-	-	Any	√	√	-	√	280	33	M	4.2* (0.9**)	4.0	USA
Michaels (2019) [42]	71.1 (14.1)	810 (49)	-	-	645 (39)	-	-	√	√	-	UC	1653	100	M	-	1.0	USA
	71.1 (14.1)	1226 (49)	-	-	976 (39)	-	-	√	√	-	UC	2503	777	M	-	1.0	USA
Scrutinio (2022) [34]	60.0 (-)	14 (25)	-	-	-	-	HFrEF	-	√	√	-	54	16	C	2.6*	2.5	Italy
Simpson (2020) [35]	65.0 (12.0)	1654 (21)	5198 (66)	1418 (18)	394 (5)	866 (11)	HFrEF	√	√	√	-	7876	1453	M	2.3	1.0, 2.0	Scotland
Spinar (2019) [43]	64.0 (12.0)	207 (19)	-	-	-	-	HFrEF	√	√	√	UC	1088	162	M	2.0*	2.0	Czech Republic
Wedel (2009) [44]	72.5 (7.1)	836 (25)	-	-	-	-	HFrEF	√	√	-	√	3342	934	M	-	-	Sweden
Welsh (2018) [45]	69.7 (11.4)	760 (41)	1260 (68)	0 (0)	167 (9)	426 (23)	HFrEF	√	√	-	√	1853	769	M	2.3	-	Scotland

Follow-up figures for Dunlay (2009) [32] were only reported as range: 3.2 years

*Abbreviations*: *ACEi* Angiotensin-converting enzyme inhibitor, *C* Composite outcome, *M* All-cause mortality, *CHF* Chronic heart failure, *HFrEF* Heart failure with reduced ejection fraction, *IQR* Interquartile range, *MRA* Mineralocorticoid receptor antagonist, *SD *Standard deviation, *UC* Unclear, √ Yes

^*^Mean reported instead of median

^**^IQR estimated using formula IQR = SD × 1.35

The added value of BNP and/or NT-proBNP was investigated for 10 base models across the 14 studies included, with the SHFM [11] being the most frequently used base model (5 studies). Appendix 5 reports factors included in the base models. Twenty model updates arose from considering more than one prediction horizon or investigating the independent incremental value of the natriuretic peptides or a logarithmic transformation of these. Of the 20 updates, 7 resulted from adding BNP or transformed BNP, and the remainder used NT-proBNP or transformed NT-proBNP (Table 4).

**Table 4:** Reported performance measures

Publication	Base model	Biomarker added to base model	Events, n	Discrimination			Risk reclassification	
				C-statistic	C-statistic’s 95% CI	Change in c-statistic after model update	IDI	NRI
AbouEzzedine (2016) [36]	SHFM	-	75	0.75	0.71–0.79	-	-	-
	SHFM	logBNP		0.78	-	0.03	-	-
Arzilli (2018) [37] (a)	3C-HF	-	198	0.741^*^	-	-	-	-
	3C-HF	lnNT-proBNP		0.78^*^	-	0.039^**^	0.031 (0.013–0.075)	0.351 (0.115–0.587)
Arzilli (2018) (b)	SHFM	-	75	0.79	0.737–0.844	-	-	-
	SHFM	lnNT-proBNP		0.818	0.766–0.869	0.028	0.042 (0.015–0.068)	0.582 (0.352–0.812)
Dunlay (2009) [32]	Clinical 1	-	122	0.757	-	-	-	-
	Clinical 1	BNP		0.789	-	0.032	-	-
Dupuy (2019) [38]	Clinical 2	-	63	-	-	-	-	-
	Clinical 2	NT-proBNP^*^		-	-	-	-	-
Ky (2011) [39]	SHFM	-	267	0.81	0.77–0.85	-	-	-
	SHFM	NT-proBNP		0.83	-	0.02	-	-
Ky (2012) [40]	SHFM	-	137	0.761	0.708–0.813	-	-	-
	SHFM	log2BNP^*^		0.809	0.763–0.854	0.048	-	0.121
May (2007) [33] (a)	SHFM	-	2142	0.71	0.66–0.76	-	-	-
	SHFM	BNP		0.78	0.73–0.82	0.07	-	-
May (2007) (b)	SHFM	-	2142	0.69	0.65–0.74	-	-	-
	SHFM	BNP		0.73	0.69–0.78	0.04	-	-
McGranaghan (2020) [41]	Clinical 3	-	33	0.84	-	-	-	-
	Clinical 3	NT-proBNP		0.86	-	0.02	-	-
Michaels (2019) [42] (a)	MAGGIC	-	100	0.664	0.644–0.684	-	-	-
	MAGGIC	BNP		0.668	0.652–0.684	0.004	-	-
Michaels (2019) (b)	MAGGIC	-	777	0.784	0.74–0.83	-	-	-
	MAGGIC	NT-proBNP		0.82	0.78–0.85	0.036	-	-
Scrutinio (2022) [34]	MAGGIC	-		0.783	0.698–0.868	-	-	-
	MAGGIC	logNT-proBNP	16	0.799	0.709–0.9	0.016	-	-
Simpson (2020) [35] (a)	Clinical 4	-	1453	0.70	-	-	-	-
	Clinical 4	NT-proBNP		0.71	-	0.01	0.005	0.136
Simpson (2020) (b)	Clinical 4	-	1453	0.69	-	-	-	-
	Clinical 4	NT-proBNP		0.70	-	0.01	0.011	0.152
Spinar (2019) [43]	Clinical 5	-	162	0.773	-	-	-	-
	Clinical 5	NT-proBNP^*^		0.79	-	0.017	0.02	0.33 (cfNRI)
Wedel (2009) [44]	Clinical 6	-	934	0.667	-	-	-	-
	Clinical 6	NT-proBNP*		0.719	-	0.052	-	-
Welsh (2018) [45]	Clinical 7	-	769	0.669	0.651–0.688	-	-	-
	Clinical 7	NT-proBNP		0.713	0.694–0.732	0.044	-	-

Some studies are described over multiple lines if they have investigated the added-value of BNP/NT-proBNP in multiple models and/or to the same model over different prediction horizons. “Clinical” describes a model not previously reported in the literature. Appendix 5 shows the predictors included in each model. Table 3 shows details of the outcomes used for number of events in each study

*Abbreviations*: *BNP* Brain natriuretic peptide, *IDI* Integrated discrimination improvement, *MAGGIC* Meta-analysis Global Group in Chronic Heart Failure, *NRI* Net reclassification index, *cfNRI* Continuous free net reclassification improvement, *NT-proBNP* N-terminal pro-brain natriuretic peptide, *SHFM* Seattle Heart Failure Model

^*^Biomarker added to base model in combination with other predictors

^**^Change in area under the receiver operating characteristic curve reported when change in c-statistic not available

All 14 studies reported model discrimination, mostly in terms of the c-statistic, with only 5 (36%) consistently reporting a confidence interval for all model updates. Two studies reported model calibration: a bar chart of expected and actual outcomes [36] and the Hosmer-Lemeshow test [37]. Although more frequently available than calibration reporting, risk reclassification data were sporadic, with NRI and IDI available for only 5 (25%) models (Table 4). Owing to the heterogeneity in the data presented in the included studies, pooling performance data for meta-analysis was not possible.

**Fig. 2:**
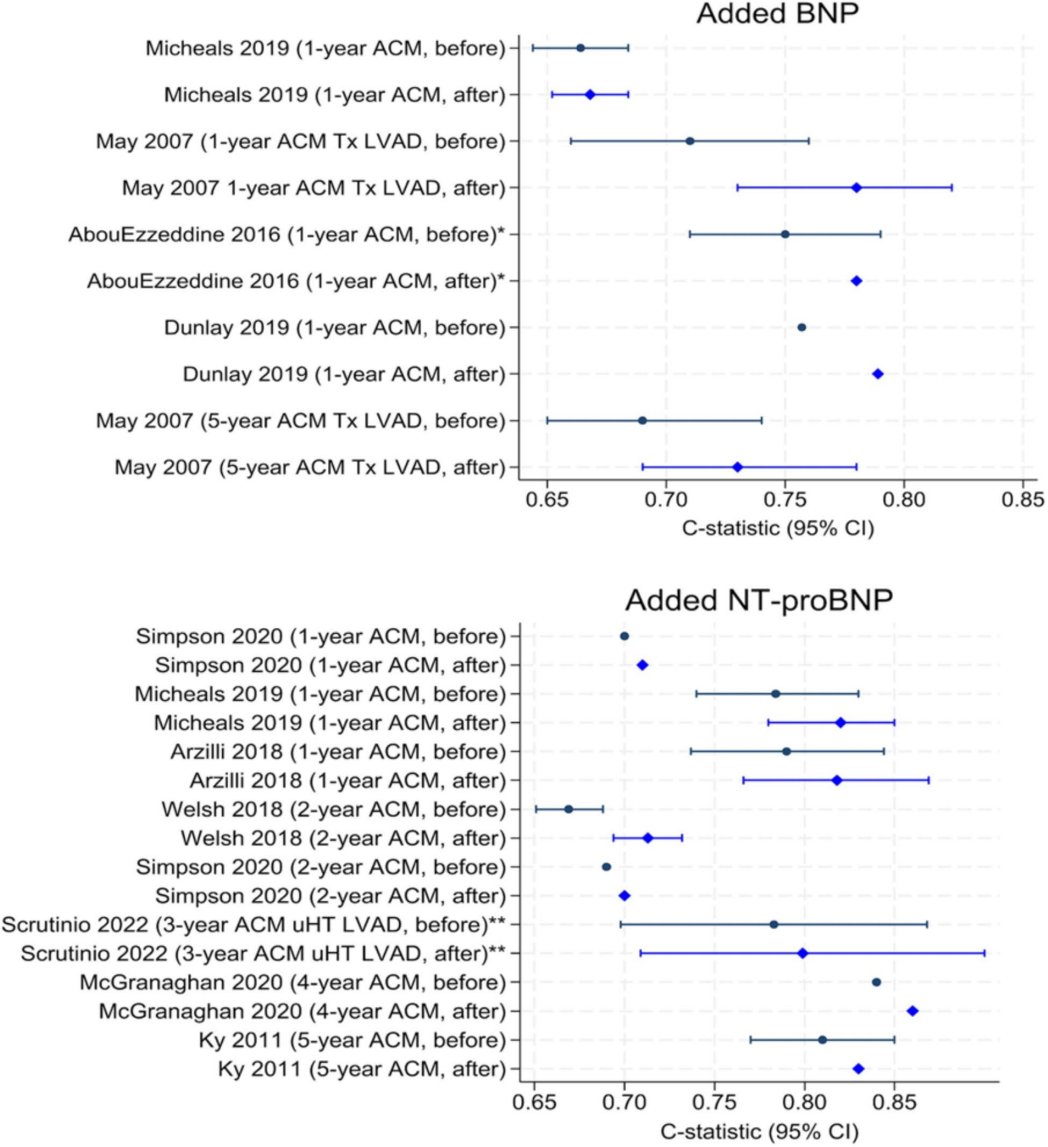
C-statistic and 95% confidence intervals of models before and after adding BNP or NT-proBNP. C-statistic and 95% confidence intervals, when available, of base models before (dots) and after (diamonds) adding BNP or NT-proBNP. Arzilli (2018) [37] figures reported for SHFM update. Abbreviations: ACM, all-cause mortality; BNP, B-type natriuretic peptide; CI, confidence interval; LVAD, left ventricular assist device; NT-proBNP, N-terminal proBNP; Tx, heart transplant; uHT, urgent heart transplant. * and ** denote transformations of BNP or NT-proBNP, respectively, added to the base model

Figure 2 presents the change in c-statistic for the studies reporting results for the addition of BNP or NT-proBNP alone. Studies reporting the addition of either of these markers in combination with other factors were not included here. It is consistently shown that the c-statistic increases in value after BNP or NT-proBNP was incorporated into the models by a range of 0.0036 [35] to 0.07 [33]. However, there was often uncertainty in the estimation of the c-statistic as indicated by the width of the 95% confidence intervals. The only study for which the intervals do not overlap is Welsh 2018 [45], but a formal method should be employed to test the statistical significance of the incremental values in general.

### Missing data

For the vast majority of studies, the change in c-statistic had to be calculated, and it was not possible to derive confidence intervals for these figures from the available information, meaning the precision of the estimated changes was unknown. The confidence interval for at least one c-statistic reported was missing for 12 (60%) out of the 20 model updates described. Regarding risk reclassification data, 14 (70%) studies did not report any measure for this. Reporting on the calibration of models was inconsistent and only present in two (14%).

### Risk of bias assessments

**Fig. 3:**
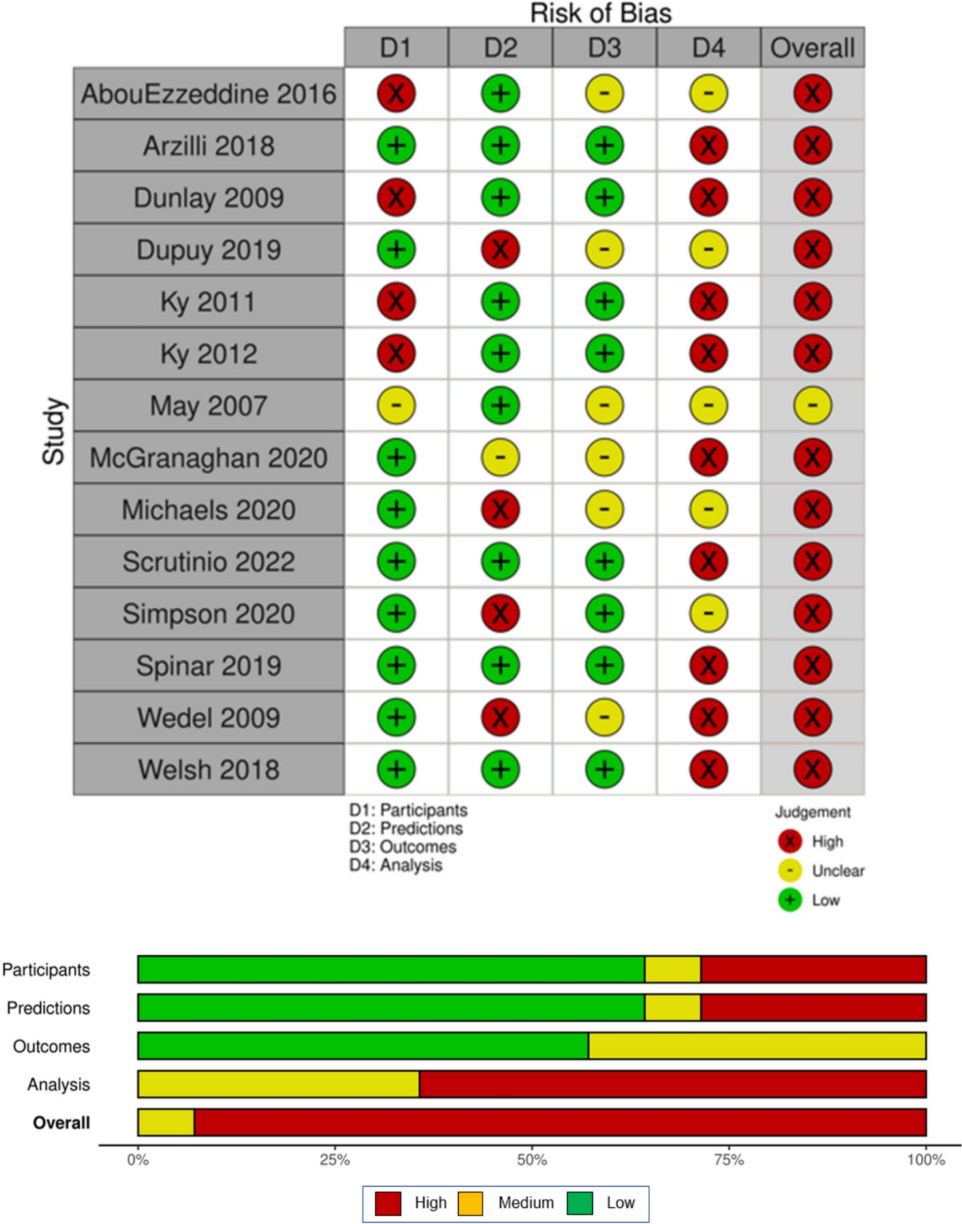
Risk of bias plots. The figure shows the risk of bias ratings of each study within each of the four PROBAST domains and overall. The horizontal bar plot shows the proportion of studies achieving each risk of bias rating overall and within the above domains

**Fig. 4:**
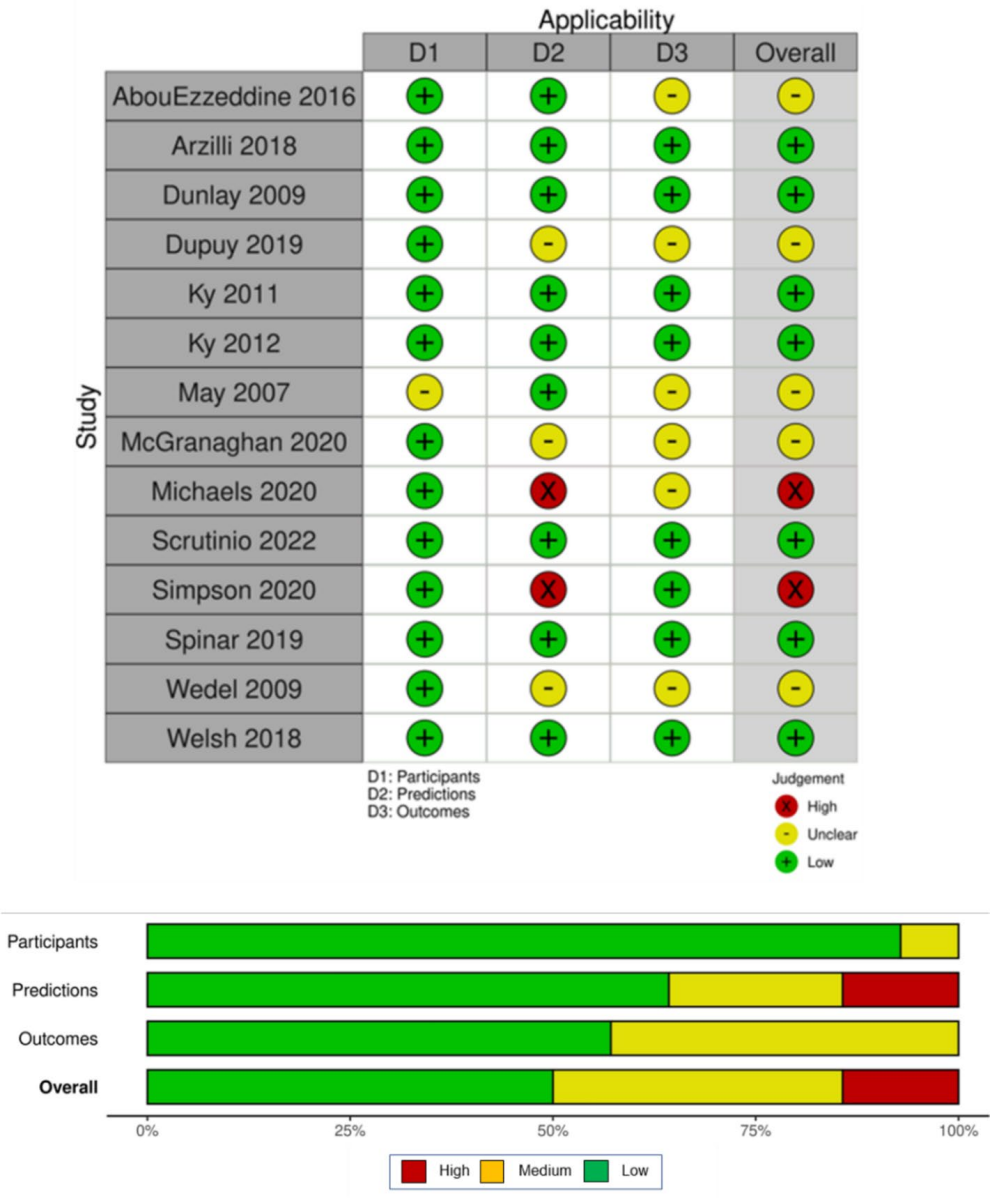
Applicability plots. The figure shows the applicability ratings for each study. Ratings are allocated overall and within the three PROBAST. The horizontal bar plot shows the percentage of studies achieving each applicability rating overall and within each domain

Figures 3 and 4 depict the risk of bias and applicability plots, respectively. All studies apart from May 2007 [33] were deemed to be at high risk of bias for reasons including lack of reporting on measures of both discrimination and calibration and excluding patients on the grounds of missing data. The research question applicability concern ratings, on the other hand, showed 7 (50%) studies to be low, with only Michaels (2019) [42] and Simpson (2020) [35] rated as high. In both cases, this was due to concerns over the “definition, assessment, or timing of predictors in the model not matching the review question” [23], for example, the NT-proBNP values being split into categories rather than being left as a continuous variable [35]. Protocols were published prior to the study being carried out in 9 (64%) of the included studies [36, 37, 39–43, 46, 47].

## Discussion

This systematic review assessed the effect of adding BNP or NT-proBNP as prognostic factors to the predictive performance of multivariable CHF models that prognosticate mortality risk. It was not possible to conduct a random-effects meta-analysis due to the heterogeneity of the pooled data from the included studies and missing performance data; therefore, results were reported narratively. Both BNP and NT-proBNP consistently improved the predictive performance of HF prognostic models in terms of discrimination; however, reporting of results in the literature lacked information regarding the statistical significance of the change in c-statistic, meaning that the possible interpretation of this information and translation into clinical practice is limited. Measures of risk reclassification (NRI and IDI) were scarcely reported. Calibration reporting was also poor, and the vast majority of studies were deemed to be at high risk of bias.

### Comparison with other studies

This review adds to the current literature by showing that with the available data, even a relatively specific set of inclusion criteria did not allow for a meta-analysis due to heterogeneity and reporting limitations among the primary studies.

A similar review, published in 2014 [22], also found meta-analysis to be unfeasible despite assessing broader outcomes including mortality and morbidity, as did another study looking at acute decompensated HF models and the addition of natriuretic peptides [48]; however, these authors concluded that BNP or NT-proBNP consistently improved prediction model performance for mortality. One HF prognostication study did manage to perform meta-analysis [49]. That paper was concerned with high-sensitivity troponin T and demonstrated that it remained independently associated with all-cause mortality when added to a prognostic model. More recently, a systematic review of prognostic models for HF with preserved ejection fraction concluded that future studies similar to those included in their review would benefit from improved methods in model development, including using PROBAST, and from externally validating models to inform readers of the models’ generalisability [50]. These authors were also unable to conduct a meta-analysis due to poor consistency in the available data.

Authors of a 2013 review of mortality risk prediction model performance in HF patients also found themselves focusing on SHFM, much like in this paper, as this was more heavily investigated than other available models and at the time reported that the discrimination and calibration left room for improvement [51].

The independent prognostic value of NT-proBNP has been reported, with a recent cohort study finding that patients with increased baseline NT-proBNP were at significantly higher risk of HF-related hospitalisation or death, independent of age, the presence of atrial fibrillation, or HF phenotype [52]. Conversely, the disadvantages of using NT-proBNP in CHF mortality prognostication — namely its varying normal ranges depending on patient age and gender, and its half-life — were found in a 2025 paper assessing the use of the biomarker systemic immune-inflammatory index (SII) for mortality prediction in HF with reduced ejection fraction (HFrEF). In this study of 521 outpatients with HFrEF, SII was modestly predictive of all-cause mortality (*AUC* 0.602, 95% *CI* = 0.531–0.673), suggesting other biomarkers might help refine survival prediction beyond NT-proBNP alone [53].

Our review finds that more methodologically consistent research evaluating the added value of natriuretic peptides to prognostic models is needed to confirm the accuracy of the models for use in clinical practice.

### Strengths and limitations

We have produced a thorough, up-to-date, sensitive, and specific systematic review of the literature pertaining to the added value afforded by the incorporation of BNP and/or NT-proBNP into CHF mortality prognostic models. However, the lack of meta-analysis hampered our ability to draw quantitative conclusions that could contribute to advancing clinical practice.

Where data values were missing, authors of the respective research were contacted to ask for this information. No response was received. Consistently, 95% CI or similar values were not available for estimates of improvement in model discrimination measured by the change in c-statistic. Risk reclassification measures, used to formally assess the improvement in model performance, were underreported in our study.

In terms of population characteristics and model predictor measurements within individual studies, missing values were generally poorly reported, with very few studies giving details of how missing data were handled (ideally through multiple imputation). We also had to estimate the median and interquartile range for some studies’ follow-up time and used them as a proxy for the prediction horizon.

Assessment of studies’ risk of bias and applicability using PROBAST was important as it gave context to the results of each study. The drawback of this tool’s thoroughness, however, and the strict criteria for marking a study as being at high risk of bias (only one domain need be high risk to render the whole study also high risk), is that it can make sensitivity analysis difficult: here, all the studies aside from one, which was marked as unclear, would have been excluded from a sensitivity analysis if a meta-analysis had been carried out.

Despite more than half of the studies having prospectively registered protocols, we were not able to pool the data and therefore were unable to formally assess any potential publication bias.

### Policy implications and future research recommendations

Natriuretic peptides are known to have predictive value, but based on the current evidence about their added value to existing prognostic models, no definitive policy implications can be drawn at this time. The reported increments in model discrimination after the addition of BNP or NT-proBNP, being small and frequently lacking confidence intervals or tests for statistical significance, make very limited meaningful interpretation of the published data.

The large dataset created by pooling the studies included in this review has the potential to provide strong evidence for the incremental value of natriuretic peptides. However, a more concerted effort in terms of study design to afford the necessary homogeneity for meta-analysis, and to ameliorate the ongoing issue of wasted research [54], is needed. Despite narrowing the included studies to just those using BNP or NT-proBNP, we still encountered heterogeneity because of transformations of these values, as well as in terms of prediction horizons and the outcomes measured.

In future, to increase the utility of studies investigating the added value of natriuretic peptides to CHF mortality prognostic models, authors should focus on clarity of data presentation — including sub-group summaries and reclassification tables — and discussion of the potential clinical significance of observed effect sizes. Formulating a standardised approach to study methodology and reporting, with an emphasis on consistent calibration testing and tests for statistical significance of change in model discrimination after the addition of natriuretic peptide, with confidence intervals for all reported values, would allow for pooling of data from multiple studies and subsequent advancement of clinical practice with potential incorporation of adequately validated prognostic models.

Our review has also highlighted the need for future studies that evaluate model performance among more diverse populations, focusing particularly on the proportion of females, people of non-white ethnicity, and people with HF of different ejection fractions.

## Conclusion

This review has highlighted the need for consistency in the methods used for updating CHF mortality prognostic models. The included studies consistently report that both BNP and NT-proBNP can improve the performance of prognostic models for mortality in HF. There is uncertainty regarding the measure of improvement and how this varies across populations; however, they are not recommended for use in clinical practice. We recommend further evaluation of the incremental prognostic value of these natriuretic peptides when added to existing models, with better standardisation of methods of studies to facilitate meta-analysis and thus provide more informative summaries of prediction performance.

## Data Availability

The dataset supporting the conclusions of this article is included within the article (and its additional files). The source data from the reports of the included studies are publicly available and accessible using the search algorithms presented in Appendix 3.

## Appendix 1

The Preferred Reporting Items for Systematic Reviews and Meta-Analyses statement (PRISMA) Checklist [26].

**Table:**

**Section/topic**	**#**	**Checklist item**	**Reported on page #**
**TITLE**			
Title	1	Identify the report as a systematic review, meta-analysis, or both.	1
**ABSTRACT**			
Structured summary	2	Provide a structured summary including, as applicable: background; objectives; data sources; study eligibility criteria, participants, and interventions; study appraisal and synthesis methods; results; limitations; conclusions and implications of key findings; systematic review registration number.	2–3
**INTRODUCTION**			
Rationale	3	Describe the rationale for the review in the context of what is already known.	3–5
Objectives	4	Provide an explicit statement of questions being addressed with reference to participants, interventions, comparisons, outcomes, and study design (PICOS).	6
**METHODS**			
Protocol and registration	5	Indicate if a review protocol exists, if and where it can be accessed (e.g., Web address), and, if available, provide registration information including registration number.	6
Eligibility criteria	6	Specify study characteristics (e.g., PICOS, length of follow-up) and report characteristics (e.g., years considered, language, publication status) used as criteria for eligibility, giving rationale.	6
Information sources	7	Describe all information sources (e.g., databases with dates of coverage, contact with study authors to identify additional studies) in the search and date last searched.	6
Search	8	Present full electronic search strategy for at least one database, including any limits used, such that it could be repeated.	Appendix 3
Study selection	9	State the process for selecting studies (i.e., screening, eligibility, included in systematic review, and, if applicable, included in the meta-analysis).	6
Data collection process	10	Describe method of data extraction from reports (e.g., piloted forms, independently, in duplicate) and any processes for obtaining and confirming data from investigators.	6–7
Data items	11	List and define all variables for which data were sought (e.g., PICOS, funding sources) and any assumptions and simplifications made.	Appendix 4
Risk of bias in individual studies	12	Describe methods used for assessing risk of bias of individual studies (including specification of whether this was done at the study or outcome level), and how this information is to be used in any data synthesis.	7
Summary measures	13	State the principal summary measures (e.g., risk ratio, difference in means).	-
Synthesis of results	14	Describe the methods of handling data and combining results of studies, if done, including measures of consistency (e.g., I^2^) for each meta-analysis.	-
Risk of bias across studies	15	Specify any assessment of risk of bias that may affect the cumulative evidence (e.g., publication bias, selective reporting within studies).	7
Additional analyses	16	Describe methods of additional analyses (e.g., sensitivity or subgroup analyses, meta-regression), if done, indicating which were pre-specified.	-
**RESULTS**			
Study selection	17	Give numbers of studies screened, assessed for eligibility, and included in the review, with reasons for exclusions at each stage, ideally with a flow diagram.	8
Study characteristics	18	For each study, present characteristics for which data were extracted (e.g., study size, PICOS, follow-up period) and provide the citations.	8–9
Risk of bias within studies	19	Present data on risk of bias of each study and, if available, any outcome-level assessment (see Item 12).	10
Results of individual studies	20	For all outcomes considered (benefits or harms), present, for each study: (a) simple summary data for each intervention group and (b) effect estimates and confidence intervals, ideally with a forest plot.	9
Synthesis of results	21	Present results of each meta-analysis done, including confidence intervals and measures of consistency.	-
Risk of bias across studies	22	Present results of any assessment of risk of bias across studies (see Item 15).	10
Additional analysis	23	Give results of additional analyses, if done (e.g., sensitivity or subgroup analyses, meta-regression [see Item 16]).	-
**DISCUSSION**			
Summary of evidence	24	Summarize the main findings including the strength of evidence for each main outcome; consider their relevance to key groups (e.g., health care providers, users, and policy makers).	10–11
Limitations	25	Discuss limitations at study and outcome level (e.g., risk of bias), and at review level (e.g., incomplete retrieval of identified research, reporting bias).	12–13
Conclusions	26	Provide a general interpretation of the results in the context of other evidence, and implications for future research.	14
**FUNDING**			
Funding	27	Describe sources of funding for the systematic review and other support (e.g., supply of data); role of funders for the systematic review.	16

## Appendix 2

Post-hoc changes to protocol methods.

All references to “biomarkers” in the protocol, here mean BNP, or NT-proBNP.

“To identify studies exploring the prognostic ability and/or added-(prognostic)value of biomarkers in patients with established heart failure (HF) of any type (i.e. ischemic, non-ischemic, chronic, acute, or decompensated).”

- This review was restricted to studies investigating chronic heart failure and BNP and/or NT-proBNP only.

The outcome has been changed from a composite of any of “overall survival, or hospitalisation related to HF, or cardiac transplantation, or mechanical assist devices implantation in end-stage HF patients” to all-cause mortality or a composite containing all-cause mortality.

“We will consider any time prediction period.”

- This review only included studies with a prediction horizon of 1 year or greater.

## Appendix 3

Search strategy for Medline for the current review.

**Table:**

	Update - December 2019
# ▲	Searches
1	exp Heart Failure/
2	((heart or cardiac) adj2 failure).ti,ab.
3	1 or 2
4	Validat$.mp. or Predict$.ti. or Rule$.mp.
5	(Predict$ and (Outcome$ or Risk$ or Model$)).mp.
6	((History or Variable$ or Criteria or Scor$ or Characteristic$ or Finding$ or Factor$) and (Predict$ or Model$ or Decision$ or Identif$ or Prognos$)).mp.
7	(Decision$ and (Model$ or Clinical$)).mp. or Logistic Models/
8	(Prognostic and (History or Variable$ or Criteria or Scor$ or Characteristic$ or Finding$ or Factor$ or Model$)).mp.
9	(Stratification or Discrimination or Discriminate or c-statistic or c statistic or "Area under the curve" or AUC or Calibration or Indices or Algorithm or Multivariable).mp. or ROC Curve/
10	4 or 5 or 6 or 7 or 8 or 9
11	3 and 10
12	(case reports or comment or editorial or letter or news or "review").pt.
13	11 not 12
14	exp animals/not humans.sh.
15	13 not 14
16	limit 15 to yr="2000 -Current"
17	(2018* or 2019* or 2020*).ed,ez,yr.
18	16 and 17
	Update - February 2024
	Medline (Ovid MEDLINE® Epub Ahead of Print, In-Process & Other Non-Indexed Citations, Ovid MEDLINE® Daily and Ovid MEDLINE®) 1946 to present
1	exp Heart Failure/
2	((heart or cardiac) adj2 failure).ti,ab.
3	1 or 2
4	Natriuretic Peptide, Brain/
5	("b-type natriuretic peptide?" or "type b natriuretic peptide?" or "brain natriuretic peptide?" or "probrain natriuretic peptide?" or "natriuretic factor 32" or bnp or nt-pro-bnp or nt-probnp or ntpro-bnp or ntprobnp).ti,ab,kf.
6	4 or 5
7	Validat$.mp. or Predict$.ti. or Rule$.mp.
8	(Predict$ and (Outcome$ or Risk$ or Model$)).mp.
9	((History or Variable$ or Criteria or Scor$ or Characteristic$ or Finding$ or Factor$) and (Predict$ or Model$ or Decision$ or Identif$ or Prognos$)).mp.
10	(Decision$ and (Model$ or Clinical$)).mp. or Logistic Models/
11	(Prognostic and (History or Variable$ or Criteria or Scor$ or Characteristic$ or Finding$ or Factor$ or Model$)).mp.
12	(Stratification or Discrimination or Discriminate or c-statistic or c statistic or "Area under the curve" or AUC or Calibration or Indices or Algorithm or Multivariable).mp. or ROC Curve/
13	7 or 8 or 9 or 10 or 11 or 12
14	3 and 6 and 13
15	(case reports or comment or editorial or letter or news or "review").pt.
16	14 not 15
17	exp animals/not humans.sh.
18	16 not 17
19	(2019* or 2020* or 2021* or 2022* or 2023* or 2024*).ed,ez,yr.
20	18 and 19

## Appendix 4

List of extracted data.

- General information: author, title, publication journal and date
- Source of data: for example, existing cohort, registry data
- Participants’ information: eligibility and recruitment method, study dates, treatments received, ethnicity, age and sex distributions
- Outcomes to be predicted: definition, blinding and time of measurement
- Information on missing data
- Sample size and how it was obtained
- External validation method (including temporal, geographical, different setting, different patients)
- Information about how the predicted individual outcomes or probabilities were calculated (using the model’s equation or a web-calculator)
- Assessment of the performance of the model in an external validation sample and whether the model was updated in response, e.g. intercept recalibrated, predictor effects adjusted, or new predictors added.
- The list and distribution of predictors (that is, the mean and standard deviation, as well as the presence of missing data and/or missing predictors) reported in the validation datasets, considering those of the derivation study as the reference.
- Model performance measures: calibration and discrimination. The corresponding estimates together with their standard error, 95% confidence interval, and (if applicable) *p* values, when reported and as appropriate. For discrimination, the area under the receiver operating characteristic curve (AUC), the c-statistic (c-index or concordance statistic). For calibration, whether a calibration plot was reported, calibration slope, calibration intercept, Hosmer-Lemeshow goodness of fit test (for logistic models), and/or observed/expected outcomes ratio (O/E ratio).
- We extracted the following measures of change in prediction accuracy usually reported by added-value studies: change in c-statistic or AUC; net reclassification improvement (NRI), which is a relative measure that assesses two models’ capacities to discriminate between patients at high and low risk of an outcome [55] and integrated discrimination improvement (IDI), which is used for evaluating a marker’s ability to predict a binary outcome [56].

## Appendix 5

Predictors used in models reported.

**Table:** **Table A** Predictors used in models reported in the included studies

	Age	Sex	Race	Region/clinical site	BMI/weight	NYHA class	EF	HF duration	Heart rate	Blood pressure	Smoking	COPD	Aetiology	Cardiac pathology	Diabetes mellitus	Vascular pathology
AbouEzzedine 2016	√	√				√	√						I			
Arzilli 2018 (a)	√					√	√			√				A, V	√	
Arzilli 2018 (b)	√	√				√	√			√			I			
Dunlay 2009	√			√	√	√				√						
Dupuy 2019	√					√	√									
Ky 2011	√	√	√		√		√			√	√		C			
Ky 2012	√	√			√	√	√			√						
May 2007	√	√				√	√						I			
McGranaghan 2020	√	√			√	√	√	√		√	√			M	√	Ca
Michaels 2019	√	√			√	√	√			√	√^2^	√			√	
Scrutinio 2022		√			√	√	√	√		√	√	√			√	
Simpson 2020	√	√	√	√	√	√	√	√	√	√					√	P
Spinar 2019	√					√										
Wedel 2009	√	√			√	√	√		√	√			I	M	√	√^4^
Welsh 2017	√	√	√	√	√	√	√	√		√		√			√	
Total/15	14	11	3	3	9	14	13	4	2	11	4	3	5	2	7	3

	Renal dys-/function	Revascularisation	ICD/PPM	ACEi or ARB	Beta blocker	Digoxin	No RAS inhibitor	Diuretics	Statin	Allopruinol	Lipids	Sodium	Uric acid	Hb/anaemia	Lymphocytes
AbouEzzedine 2016	Cr		√	√	√^1^	√		√	√			√			
Arzilli 2018 (a)	√				√		√							√	
Arzilli 2018 (b)								√	√		√	√	√	√	√
Dunlay 2009	Cr											√			
Dupuy 2019															
Ky 2011	Cr		√	√	√										
Ky 2012				√				√	√	√					
May 2007	Cr		√	√	√^1^	√		√	√			√			
McGranaghan 2020	Cr				√						√				
Michaels 2019	Cr			√	√										
Scrutinio 2022	Cr				√		√								
Simpson 2020		√					√^3^								
Spinar 2019								√				√	√	√	
Wedel 2009	Cr	√^5^	√												
Welsh 2017				√	√										
Total/15	8	2	4	6	8	2	3	5	4	1	2	5	2	3	1

Abbreviations: *A* Atrial fibrillation, *ACEi* Angiotensin converting enzyme inhibitor, *ARB *Angiotensin receptor blocker, *BMI* Body mass index, *C* Cardiomyopathy, *Ca* Carotid artery disease, *COPD* Chronic obstructive pulmonary disease, *Cr* Serum creatinine, *EF* Ejection fraction, *Hb* Haemoglobin, *HF* Heart failure, *I* Ischaemic, including ischaemic cardiomyopathy, *ICD* Implantable cardioverter defibrillator, *M* Myocardial infarction, *NYHA* New York Heart Association functional classification, *P* Peripheral artery disease, *PPM* Permanent pacemaker, *RAS* Renin-angiotensin system inhibitor, *V* Severe valve disease, √ Yes

^1^Beta blocker includes carvedilol

^2^Current smoker

^3^Specifically no sacubitril or valsartan

^4^Includes intermittent claudication, stroke, aortic aneurysm

^5^Includes percutaneous coronary intervention, and coronary artery bypass graft. Lipids include hyperlipidaemia, cholesterol, triglycerides, low density lipoprotein. Total is out of 15 base models investigated (different to the 10 base models mentioned in the main text due to multiple studies investigating the same model)

## Abbreviations

AUC: Area under the receiver operating characteristic curve
BNP: B-type natriuretic peptide
CHARMS: Checklist for critical Appraisal and data extraction for systematic Reviews of prediction Modelling Studies
CHF: Chronic heart failure
CI: Confidence interval
cfNRI: Continuous free net reclassification improvement
HF: Heart failure
HFrEF: Heart failure with reduced ejection fraction
IDI: Integrated discrimination improvement
LVAD: Left ventricular assist device implantation
MAGGIC: Meta-analysis Global Group in Chronic Heart Failure
NT-proBNP: N-terminal proBNP
NRI: Net reclassification index
PICOTS: Population, Intervention, Comparator, Outcome, Timing, and Setting
PRISMA: Preferred Reporting Items for Systematic reviews and Meta-Analyses statement
PROBAST: Prediction model risk of bias assessment tool
SD: Standard deviation
SHFM: Seattle Heart Failure Model
SII: Systemic immune-inflammatory
